# P-1359. Epidemiology of Carbapenem Resistant Enterobacterales in Monroe County, NY 2016-2023

**DOI:** 10.1093/ofid/ofae631.1536

**Published:** 2025-01-29

**Authors:** Rebecca Tsay, Jenna Dietz, Christina B Felsen, Ghinwa Dumyati

**Affiliations:** New York Rochester Emerging Infections Program at the University of Rochester Medical Center, Rochester, New York; Center for Community Health and Prevention, Rochester, New York; University of Rochester, Rochester, NY; New York Emerging Infections Program and University of Rochester Medical Center, Rochester, New York

## Abstract

**Background:**

Carbapenem-resistant Enterobacterales (CRE) infections pose an urgent threat due to the limited treatment options and the potential for resistance spread among carbapenemase producing (CP) CRE strains.

**Figure:**
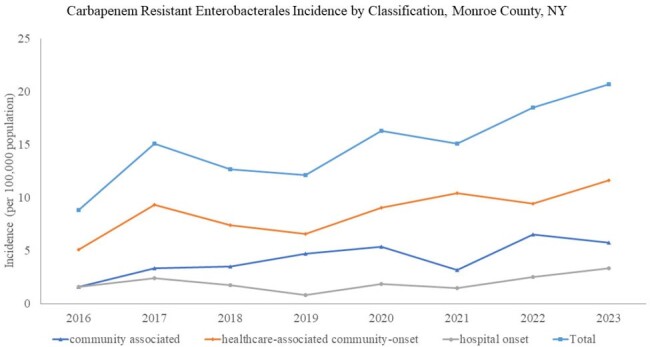

**Methods:**

We conducted active population-based surveillance for select Enterobacterales species resistant to ≥ 1 carbapenem from a normally sterile site or urine in Monroe County, NY as part of the CDC Emerging Infections Program. Epidemiologic data were collected through medical record abstraction. Cases were classified as hospital onset (HO) if they occurred 72 hours after hospital admission, healthcare-associated community-onset (HACO) cases if they had a prior healthcare risk factor, or community associated (CA). CRE isolates were tested at CDC or Northeast Antimicrobial Resistance Laboratory Network.

**Figure:**
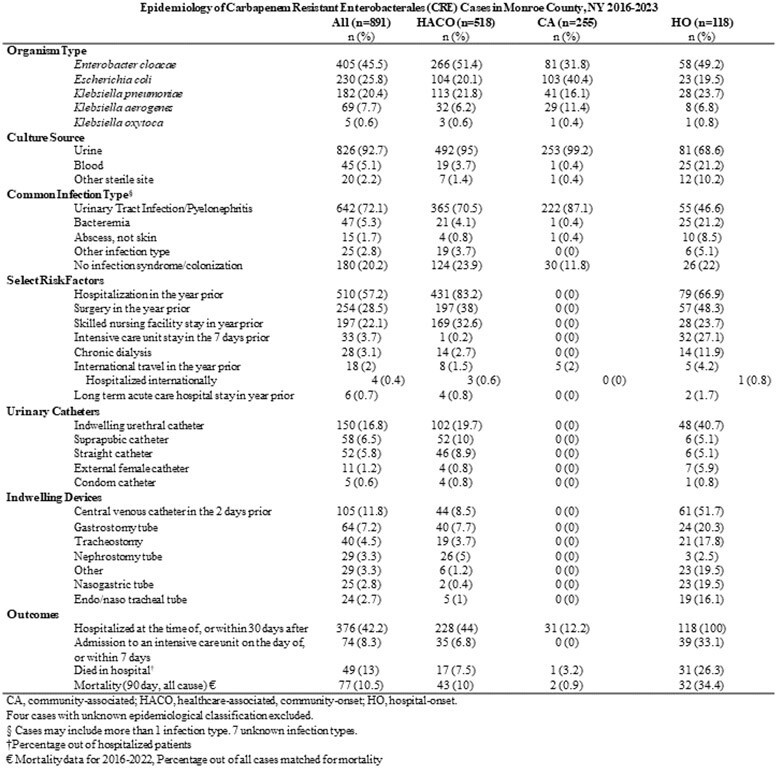

**Results:**

From 2016-2023, CRE incidence increased 135% from 8.8 to 20.7 per 100,000 population with 895 incident CRE cases identified in 745 unique patients. Patients were mostly non-Hispanic (89.2%), White (82.1%) and female (67.8%). Urine was the most common source (92.7%). 58.1% of cases were HACO, 28.6% CA and 13.2% HO. Urinary tract abnormalities (58%) and recurrent UTIs (32.6%) were prevalent. Prior healthcare exposures including hospitalization (57.2%), surgery (28.5%), and skilled nursing home stay (22.1%) were common in healthcare associated cases. Few (2%) had a history of international travel. Hospital onset cases were more likely to have CRE isolated from blood (21.2%), require ICU admission (33.1%), and die within 90 days of their infection (34.4%). 272 CRE isolates underwent carbapenemase testing, of those, 78 (28.7%) were CP-CRE: 71.1% *bla*_KPC_, 19.3% *bla*_NDM_, and 9.6% *bla*_OXA48_ with documented emergence of *bla_NDM_* CP in 2021. Only 6 (9.5%) of CA isolates were CP-CRE compared to 18 (40%) of HO, and 54 (33.1%) of HACO.

**Figure:**
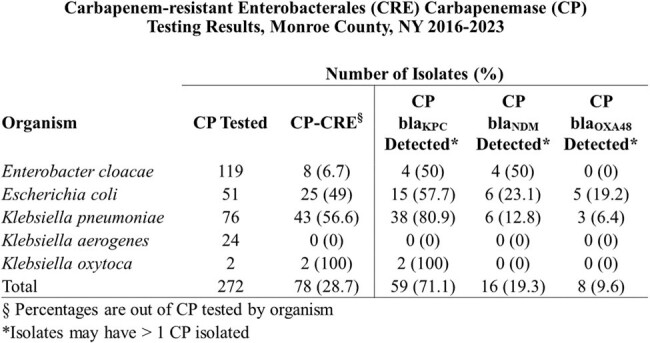

**Conclusion:**

The incidence of CRE in Monroe County, NY has significantly increased since 2016 with documented emergence of *bla_NDM_*-producing strains. The high proportion of healthcare-associated CRE cases, particularly CP-CRE, underscores the need for enhanced infection control measures and ongoing surveillance. Further investigation is needed into the risk of CP-CRE in CA cases.

**Disclosures:**

**All Authors**: No reported disclosures

